# Patient and Public involvement to understand and inform the co-design of how we communicate mortality risk for patients aged 90 and over considering elective total hip replacement (THR)

**DOI:** 10.1186/s40900-026-00900-w

**Published:** 2026-05-04

**Authors:** Ravi Patel, Ben Woodhouse, Amr Selim, Robin Banerjee, Niall Steele, Edward Dickenson, Rajpal Nandra, Sarah L. Whitehouse, Adele Higginbottom, Johnathan T. Evans, Geraint Thomas

**Affiliations:** 1 Department of Trauma and Orthopaedics, The Robert Jones and Agnes Hunt Orthopaedic Hospital, Oswestry, UK; 2 Centre of Regenerative Medicine Research, The School of Pharmacy and Bioengineering, Keele University, Staffordshire, UK; 3 School of Medicine, Keele University, Keele, UK; 4 Queensland University of Technology, Brisbane, QLD Australia; 5 Impact Accelerator Unit, Keele University, Keele, Staffordshire, ST5 5BG UK; 6 Exeter Hip Unit, Princess Elizabeth Orthopaedic Centre, Royal Devon and Exeter NHS Foundation Trust, Exeter, UK; 7 Exeter Medical School, University of Exeter, St Lukes Campus, Exeter, UK; 8 National Institute for Health and Care Research Exeter Biomedical Research Centre, Royal Devon University Healthcare NHS Foundation Trust and University of Exeter, Exeter, UK; 9 NIHR Academic Clinical Fellow Robert Jones and Agnes Hunt Orthopaedic Hospital Gobowen, Oswestry, SY10 7AG UK

**Keywords:** Patient and Public involvement, Risk, Communication, Shared decision-making, Total hip replacement, Total hip arthroplasty, Nonagenarian

## Abstract

**Background:**

Patient and Public Involvement and Engagement (PPIE) is integral to ensuring health research remains grounded in patient priorities and lived experience. This is especially critical for underrepresented groups, such as adults aged 90 and over considering elective total hip replacement (THR). This demographic experiences a small but meaningful risk of postoperative mortality, yet conventional numeric risk communication often fails to align with their values, communication preferences, and the existential context of very advanced age. Traditional PPIE methods, like focus groups, may inadvertently exclude this group due to sensory, mobility, or digital barriers.

**Methods:**

This PPIE activity engaged eight public contributors aged 90–96 years with lived experience of total hip replacement or caring for someone who underwent it (including two carers) through semi-structured one-to-one telephone conversations, aligned with the GRIPP2 Short Form and UK Standards for Public Involvement. We detail our approach to building rapport and facilitating nuanced conversations remotely, focusing on three domains: research context, communication preferences, and decision-making. Our methodology was designed to prioritise relationship-building and flexibility to overcome barriers to participation.

**Results:**

Inductive review of facilitator notes identified three central themes:

1. A Pragmatic Conceptualisation of Risk: Participants framed mortality risk as an acceptable “chance you take,” shaped by life experience and advanced age.
2. Values-Based Decision-Making: Quality of life outcomes: mobility, independence, and maintaining an optimistic outlook were prioritised over longevity alone.
3. The Imperative for Compassionate Communication: Participants emphasised a need for clear, respectful, and multi-modal communication, while explicitly rejecting ageist or overly clinical language.

Contributors unanimously affirmed the importance of this topic, feeling that as an underserved community, their perspectives on mortality were both valuable in decision making and long overdue.

**Limitations:**

This PPIE activity involved a small number of contributors and used telephone-only engagement. While this ensured accessibility, it may limit transferability to other contexts. Future work should test alternative formats and include larger, more diverse samples to enhance generalisability to the nonagenarian populations. Nonetheless, the rich feedback insights from this unrepresented group provide a valuable foundation for future work.

**Conclusions:**

The findings challenge the primacy of numerical risk presentation for this demographic, underscoring that communication must prioritise dignity, optimism, and clarity. Crucially, contributors affirmed that discussing mortality is a necessary part of informed consent, countering assumptions that this topic should be avoided. These insights directly inform more sensitive and relevant research design and clinical communication tools, ensuring they are grounded in the values and priorities of the patients they are meant to serve.

## Abstract (plain language summary)

This project involved talking directly to eight contributors aged 90 and over — including people who had undergone total hip replacement (THR) and their carers — to understand their views on the risk of dying from this operation. THR can greatly improve mobility and quality of life, but it carries a small risk of death. Patients and families need this information explained clearly and sensitively. We spoke individually with each contributor by telephone. They told us that:

- They see the risk of surgery as a normal part of life.
- Improving their quality of life, by reducing pain and keeping them mobile, is more important than just living longer.
- Doctors should talk about the risks of hip replacement honestly, respectfully, and using simple words and pictures.

The contributors felt they were part of an age group that is often underrepresented and overlooked in healthcare, making their input especially valuable. Their feedback is being used to create better information leaflets and communication guides, ensuring that future patients and their families can have supportive and understandable conversations about surgery.

## Background

Patient and Public Involvement and Engagement (PPIE) has evolved from a supplementary activity to a critical component of contemporary health research, ensuring that studies are relevant, ethical, and reflective of the priorities of those they aim to serve [1, 2]. In the UK, the National Institute for Health and Care Research (NIHR) standards for public involvement stipulate the importance of inclusive, flexible, and reflective practices, particularly for groups traditionally underrepresented in research [3, 4]. This is crucial to avoid over-representing the perspectives of those who are more affluent, digitally literate, or physically able to participate in traditional methods like focus groups.

Adults aged 90 years and over represent one of the most rapidly growing and often underserved demographics in orthopaedic research. Elective total hip replacement (THR) can offer this group profound improvements in pain, mobility, and quality of life. However, it also carries a small but significant postoperative mortality risk of approximately 2% within 90 days, according to the UK National Joint Registry [5, 6]. While the statistical risk is known, there is a critical lack of understanding regarding *how* this risk is perceived, valued, and preferred to be communicated by the patients themselves. Communicating this risk transparently and sensitively is a complex challenge for clinicians, patients, and families alike [7, 8]. For this underserved community, who felt their age group was frequently overlooked in healthcare planning, the very act of investigating this topic was later identified by participants as being of significant personal and collective value.

Conventional risk communication often relies on numerical probabilities (e.g., “a 2 in 100 chance”), which may not be cognitively meaningful or emotionally accessible for patients aged 90 and over [9]. For individuals in their tenth decade of life, mortality is not a distant abstraction but a proximate reality; its communication is imbued with personal, existential, and social meanings that numerical data alone cannot capture. Furthermore, assumptions that very old patients may wish to avoid discussions about mortality can lead to paternalistic communication or the omission of risk information altogether, undermining informed consent and shared decision-making.

Traditional PPIE methods, such as sessional focus groups with agendas set by researchers, present significant barriers for this population. These can include hearing loss, fatigue, mobility limitations that prevent travel, and a lack of familiarity with digital meeting platforms [14, 15]. Consequently, their perspectives are often absent from the very research intended to benefit them, perpetuating a cycle of exclusion and potentially misaligned clinical interventions [10, 11].

We therefore designed this PPIE activity with inclusivity and nuance as its core principles. It is important to distinguish this activity from the subsequent fellowship qualitative study it was designed to inform: the PPIE activity was not itself a research study generating generalisable findings, but a structured involvement exercise with three specific aims. First, to explore with adults aged 90 and over and their carers how mortality risk associated with elective THR is conceptualised and preferred to be communicated. Second, to validate the relevance of the proposed fellowship study’s aims from the perspective of those it seeks to benefit. Third, to co-design study materials — including patient-facing information and the interview topic guide — that are sensitive, accessible, and grounded in patient-identified priorities. Rather than seeking a consensus view, we aimed to capture a heterogeneous range of perspectives reflecting the diversity of experience within this population.

## PPIE methods

### Design and ethical framework

This work was conducted as a structured PPIE activity to guide research design, in accordance with the UK Standards for Public Involvement [3] and reported using the GRIPP2 Short Form [12]. This PPIE activity was a precursor to an NIHR-funded fellowship qualitative study examining how mortality risk is communicated to patients aged 90 and over considering elective THR. The specific aims of this PPIE activity were to: (i) validate the relevance and acceptability of the proposed study aims with older adults and carers; (ii) co-design patient-facing communication materials including language, format, and tone; and (iii) directly inform the interview topic guide for the subsequent qualitative study. The objective was therefore to gather insight and guidance to *inform* a future qualitative study, rather than to generate generalisable findings through formal data analysis. The discussion guide was reviewed by a clinical and PPI advisory panel (comprising clinical colleagues and a pre-existing PPI group established for the fellowship study) prior to use. While formal ethical review was not required for this involvement activity, the principles of the Declaration of Helsinki were rigorously upheld. This included obtaining verbal informed consent, ensuring confidentiality, guaranteeing voluntary participation, and providing honoraria in recognition of contributors’ time and expertise. Given the age and vulnerabilities of contributors, safeguarding considerations were observed throughout: all conversations were conducted sensitively, with the facilitator trained to recognise signs of distress, and participants were reminded of their right to withdraw at any point. Capacity and fatigue were monitored informally throughout each conversation, with the facilitator checking in on participants’ comfort and offering to pause or end the call if needed.

### Participant recruitment and characteristics

We purposively recruited eight public contributors aged 90–96 years to capture a range of experiences. The cohort comprised six patient contributors, all of whom had undergone THR after the age of 90, and two carers. The two carers were family members of older adults in this age group who had direct experience of supporting someone through the surgical decision-making process; they did not participate in place of an older adult who was unable to contribute directly, and their perspectives are distinguished where relevant in the findings. Recruitment was conducted via established clinical networks, including community geriatricians and hospital orthopaedic teams, to identify individuals who might not otherwise engage with research opportunities. In total, fourteen individuals were approached; eight agreed to participate and six declined, citing fatigue or ill health. Of the eight participants, five were female and three were male; all identified as White British. Each participant received a £25 voucher as an honorarium.

### Procedure and relational approach

Semi-structured one-to-one telephone conversations, lasting approximately 45–60 min, were conducted by the lead researcher, an NIHR Academic Clinical Fellow in Trauma and Orthopaedics. Telephone engagement was selected over in-person or digital methods as it removed barriers relating to mobility, travel, and digital literacy. However, it is acknowledged that telephone-based methods are not without limitations for this population, particularly regarding hearing loss and mild cognitive impairment, which are prevalent in adults aged 90 and over [14, 15]. To mitigate these, the facilitator assessed hearing ability at the outset of each conversation by asking participants directly whether they could hear clearly and whether they typically experienced any difficulty on the telephone. Adaptations included speaking slowly and clearly, repeating or rephrasing questions as needed, and offering to reschedule if a participant appeared fatigued. One participant required a second call due to fatigue. It is also acknowledged that telephone engagement, unlike face-to-face interaction, precludes visual cues, which may affect the facilitator’s ability to fully assess comprehension or distress; this was managed through attentive listening and regular verbal check-ins throughout each call.

The conversations were guided by a flexible discussion framework featuring fifteen open-ended questions, organised into three key domains (Table 1). However, the interaction was designed to be more than a simple data extraction exercise. We explicitly adopted a relational approach, informed by models of therapeutic communication that emphasise empathy, unconditional positive regard, and congruence [13]. This involved:

- Building Rapport: Beginning conversations with informal discussion to put participants at ease and affirm the value of their personal expertise.
- Active Listening: Allowing participants ample time to reflect and respond, acknowledging the emotional weight of the topic.
- Demonstrating Empathy: Verbally acknowledging the challenges and perspectives shared by contributors.

With verbal consent, conversations were audio-recorded solely to ensure the accuracy of the facilitator’s contemporaneous notes. All identifiable information was removed during the transcription of these notes to ensure anonymity.

**Table 1:** Aims of PPIE activities and questions asked to PPIE informants

Discussion Domain	Example Questions	Linked PPIE Objective	Intended Output / Influence on Project Design
Research Context and Importance	“What are your thoughts on researching how we discuss surgical risks with patients aged 90+?” “What should researchers prioritise in this work?”	To ensure the proposed research reflects issues considered meaningful and relevant by older adults and carers.	To explore participants’ perceptions of the value and relevance of studying mortality risk communication, and to identify priorities and expectations for future research.
Communication Approaches and Preferences	“How would you want a doctor to talk about a small but serious risk like mortality?” “What types of decision aids would be most helpful? (charts, diagrams, online tools)”	To co-design acceptable and accessible ways of communicating surgical mortality risk.	To identify preferred language, tone, and modes of presentation that promote clarity, dignity, and understanding in patient–clinician discussions.
Decision-Making and Practical Application	“What information matters most when weighing risks and benefits of surgery?” “How do personal priorities like independence or quality of life affect surgical decisions?”	To ensure study questions and materials align with patient and carer values that underpin surgical decision-making.	To explore how older adults weigh risks against quality-of-life outcomes, informing the framing of decision-support materials and qualitative study design.

### Analytical approach

A full thematic analysis was not undertaken, consistent with the reflective nature of a PPIE activity; the content presented here constitutes involvement insight rather than research data, and should be interpreted accordingly. Instead, the facilitator’s detailed contemporaneous notes were reviewed inductively. This process involved repeatedly reading the notes to identify recurring concepts, patterns of phrasing, and particularly salient insights. These elements were then synthesised into three overarching interpretive themes. To ensure accuracy, a sample of quotations was checked against the audio recordings by a second member of the research team, and contributors were offered the opportunity to review summary findings. Verbatim quotations are included to ensure contributors’ voices remain central, and were selected to be illustrative of the breadth and depth of perspectives expressed rather than to reflect any single view.

### Findings

Analysis of the discussions revealed three overarching themes that reflected how contributors conceptualised and communicated mortality risk.

### Theme 1: A pragmatic conceptualisation of risk

Participants did not engage with mortality risk as an abstract statistic but rather as a tangible, and often acceptable, part of life’s calculus. Phrases such as, “Oh, well, I know it’s there. Yes, it’s a chance you take,” were typical. For some, professional backgrounds informed this view: “I was a nurse for 45 years—there’s a risk in most things.” A sense of gratitude and acceptance born from advanced age was also evident: “Once you’ve had your 90 years, you’ve got to be thankful.” This perspective fostered a calm acceptance, with one participant stating, “No, I wasn’t worried. I just had every confidence.” Importantly, contributors consistently affirmed the value of the research topic itself. As one participant stated, “It’s high time someone asked us,” underscoring that as an underserved community, they felt this investigation was both important and long overdue.

### Theme 2: Values-based decision-making: quality of life over longevity

The decision to undergo surgery was overwhelmingly driven by the pursuit of improved quality of life, not the mere extension of life. Participants explicitly prioritised outcomes such as pain relief (“The chances of the pain going”) and functional independence (“Keeping me mobile”). An optimistic outlook was a recurring motif: “I’m an optimist,” and “Life’s to be lived, isn’t it?” This value set was powerfully summarised by one participant who, reflecting on their experience, said, “If I had the choice, I’d go through it again.”

### Theme 3: The imperative for compassionate communication

**Fig. 1:**
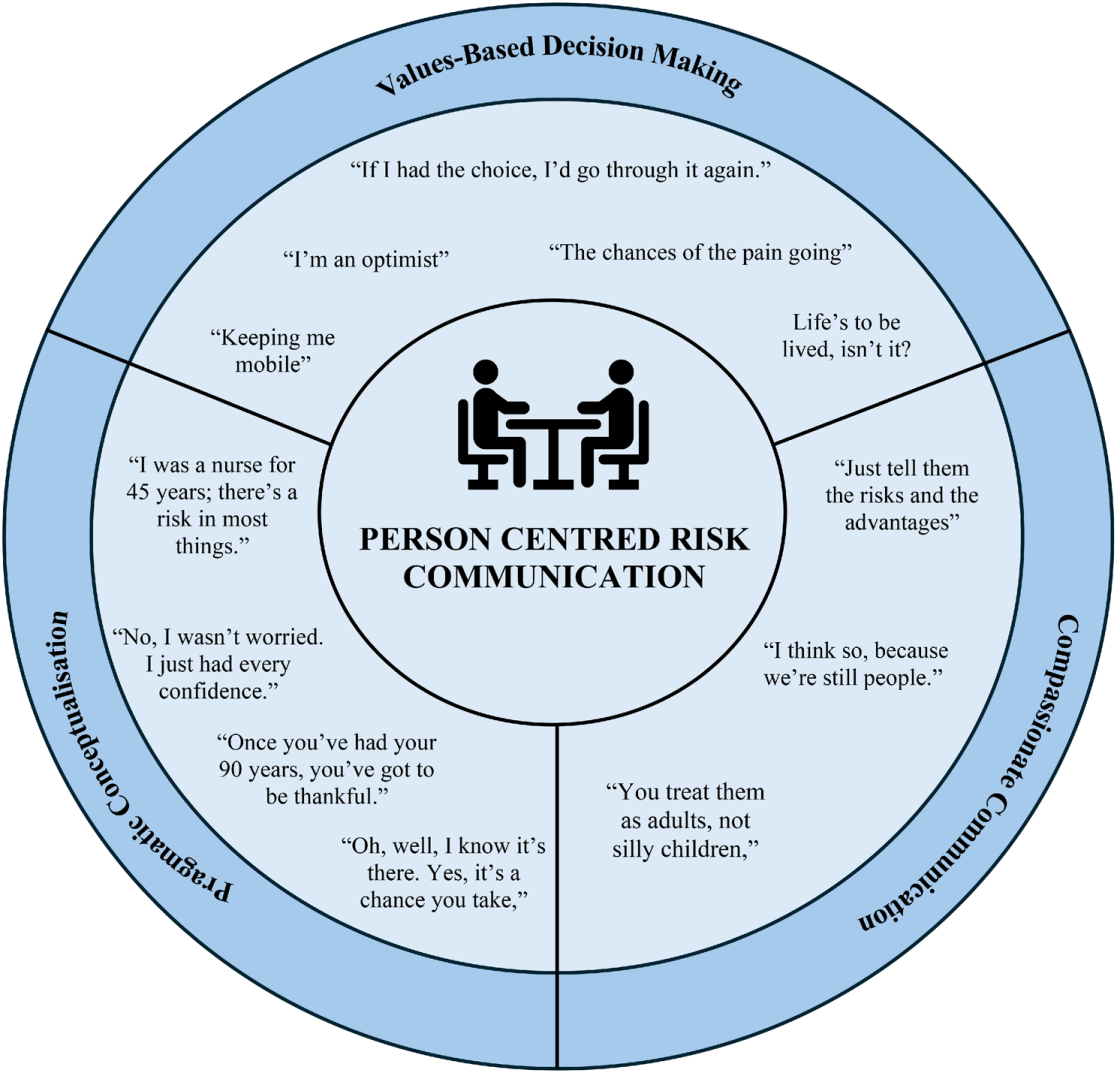
Infographic summarising the three core themes and selected illustrative quotes from contributors, designed for use in patient-facing materials

Participants expressed clear preferences for how risk should be communicated. They valued honesty and clarity (“Just tell them the risks and the benefits”) but within a context of profound respect. They rejected paternalism, with one contributor emphasising, “You treat them as adults, not silly children,” and another affirming, “I think so, because we’re still people.” In terms of format, a multi-modal approach was favoured, combining simple language (“Words”) with visual aids (“Diagrams always help”) and short videos. Figure 1 presents an infographic developed as a direct output of this PPIE activity, designed for use in patient-facing materials rather than as a restatement of the findings above; it illustrates the themes in the accessible, visual format that contributors themselves recommended.

### PPIE impact statement

This activity had direct and substantive impact on the fellowship project, directly addressing the aim of co-designing study materials and informing research delivery:

1. Reframed Research Focus: The findings established that communication tools must prioritise clarity, respect, and optimism over statistical precision, directly shaping the project’s primary outcome.
2. Informed Material Development: Insights on language and multi-modal formats (e.g., short videos, diagrams) are being used to redesign patient information leaflets and the creation of a summary infographic (Fig. 1).
3. Shaped Subsequent Study Design: The themes and specific concerns raised (e.g., being treated like “silly children”) directly informed the interview schedule for the main qualitative study. Specifically, questions were rephrased to avoid clinical jargon, the order of topics was reorganised to begin with quality-of-life priorities before mortality risk, and new questions were added to explore participants’ views on communication format and tone. This ensures the qualitative study is grounded in and responsive to the values expressed by this population.
4. Established Ongoing Involvement: The activity led to the creation of a standing Public Advisory Group of older adults to provide continuous input throughout the fellowship qualitative study. Six of the eight contributors expressed interest in continued involvement. This group will be engaged at key stages of the qualitative study, including review of emerging findings, co-production of patient-facing materials, and feedback on dissemination outputs. We recognise that longitudinal involvement with this age group requires careful planning: meetings will be conducted by telephone, kept brief (no longer than 30 min), and scheduled flexibly to accommodate fluctuating health. The burden on contributors will be minimised, and the group will be supported to reduce or pause involvement without penalty if health or capacity changes.

## Discussion

This PPIE activity demonstrates that telephone-based engagement can be a valuable and accessible method for capturing the nuanced perspectives of adults aged 90 years and over, a group often excluded from digital and in-person research activities. It should be understood as one component of an inclusive toolkit, alongside home visits, postal methods, and supported digital engagement, rather than a universally inclusive approach in its own right [14, 15]. The contributors provided invaluable insights that challenge conventional clinical approaches to risk communication. They reframed surgical mortality risk from a fearful probability to a natural and acceptable aspect of life, contingent on the potential for improved quality of life.

A key finding was the participants’ affirmation of the research’s importance. By explicitly valuing the investigation into mortality communication, they highlighted a critical gap in patient-centred care for their demographic. This reinforces the necessity of PPIE not only for refining research questions but also for validating the social and ethical relevance of a study from the perspective of underserved communities.

These findings counter assumptions that patients aged 90 and over may avoid discussions about mortality. Instead, they demand such conversations be conducted with empathy, partnership, and a respect for patient autonomy. The emphasis on compassion and person-centred language over numerical detail has significant implications for both shared decision-making in clinical practice and the design of patient-facing materials.

It is worth addressing directly whether this work offers anything beyond what is already known about best practice in shared decision-making. Principles such as person-centred communication, incorporating patient values, and accessible language are indeed established standards. The specific and novel contribution of this work, however, lies not in affirming these principles in the abstract, but in demonstrating empirically how they manifest — and what they look like in practice — for one of the most underrepresented groups in surgical research: adults aged 90 and over. This population has distinct existential, cognitive, and communicative characteristics that are not reliably captured by applying insights from younger cohorts. The pragmatic acceptance of mortality, the explicit prioritisation of quality over length of life, and the rejection of paternalistic framing are not simply restatements of generic best practice; they are specific, age-contextualised preferences that challenge clinicians to move beyond standard approaches and adopt genuinely tailored communication strategies for this demographic.

A further important observation, which emerged from the findings and warrants acknowledgement, is that contributors themselves consistently framed mortality risk within a broader landscape of perioperative concern. Outcomes such as functional decline, loss of independence, failure to return home, and postoperative delirium were discussed as part of contributors’ decision-making calculus, alongside mortality. This reflects the literature on shared decision-making in older surgical patients, in which mortality is rarely the only or primary consideration [7, 8]. The focus of this PPIE activity — and of the subsequent fellowship study — on mortality risk communication is therefore intentionally bounded: it does not claim to capture the full scope of perioperative risk communication, and future PPIE and qualitative work should explicitly address how non-mortality risks are communicated to and understood by this population. This represents a meaningful direction for future research, and one that this activity has helped to identify.

### Strengths and limitations

A key strength of this work is the genuine inclusion of adults aged 90 and over, a group rarely represented in PPIE activities. The relational, telephone-based approach was tailored to the needs of this population and enabled rich, nuanced conversations that would not have been possible through conventional group formats. The use of the GRIPP2 Short Form and NIHR Standards for Public Involvement provides a transparent reporting framework. There are, however, important limitations to acknowledge. The sample was small (*n* = 8) and all participants identified as White British, which limits the transferability of findings to more ethnically diverse nonagenarian populations. The predominance of patient contributors who had undergone THR (six of six patient contributors) may have introduced a degree of selection bias towards positive surgical experiences: contributors who had elected for and survived surgery may hold more favourable views of operative risk than those who declined, were not offered surgery, or experienced adverse outcomes. Future work should deliberately recruit from across the full spectrum of surgical decision pathways. Telephone engagement, while practical, precluded visual cues and may have been challenging for those with hearing difficulties, despite the adaptations described. The absence of visual contact also has implications for the researcher-participant power dynamic: without the ability to observe non-verbal cues such as hesitation, discomfort, or fatigue, the facilitator was reliant on verbal signals alone to gauge how contributors were engaging. This may have resulted in some contributors masking uncertainty or discomfort, and is a specific limitation of remote engagement with very old adults that future PPIE studies should reflect on. The perspectives of the two carer contributors offered complementary insights but were not formally distinguished from those of patient contributors in all analytical steps; future work should explore carer-specific experiences separately. Finally, researcher positionality as a clinician conducting PPIE with patients may have introduced an inherent power imbalance. Contributors may have felt reluctant to express negative views about clinical communication in the presence of a clinical researcher, even when encouraged to speak freely. The relational approach and informal telephone format were designed to mitigate this, but cannot eliminate it entirely.

## Conclusion

This PPIE activity yielded three fundamental insights that directly challenge conventional clinical approaches to risk communication for adults aged 90 and over considering elective surgery. First, participants conceptualised mortality risk not as a frightening statistic, but as a pragmatic and acceptable trade-off for the potential to restore quality of life. Second, their decision-making was driven overwhelmingly by values centred on mobility, independence, and optimism, rather than a desire to extend life at all costs. Third, and most critically, they demanded compassionate communication that is clear, respectful, and multi-modal, firmly rejecting paternalistic language.

These findings underscore that for this demographic, effective risk communication is not about avoiding the topic of mortality, but about framing it within a context of honesty, respect, and empowerment. The participants’ clear sentiment of being an underrepresented community reinforced the critical need to actively seek and value their perspectives. By prioritising dignity and patient-identified values over numerical precision, clinicians and researchers can better partner with this growing population to support informed, values-congruent healthcare decisions. The strong endorsement from participants confirms that centring the voices of this underserved community is not only methodologically sound but also a moral and practical imperative for developing truly patient-centred care.

## Data Availability

No datasets were generated or analysed during the current study.
